# Children with idiopathic short stature have significantly different gut microbiota than their normal height siblings: a case-control study

**DOI:** 10.3389/fendo.2024.1343337

**Published:** 2024-02-23

**Authors:** Liora Lazar, Adi Eshel, Lelyan Moadi, Michal Yackobovitch-Gavan, Meytal Bar-Maisels, Biana Shtaif, Michal Nevo, Moshe Phillip, Sondra Turjeman, Omry Koren, Galia Gat-Yablonski

**Affiliations:** ^1^ School of Medicine, Faculty of Medical and Health Sciences, Tel Aviv University, Tel Aviv, Israel; ^2^ The Jesse Z and Sara Lea Shafer Institute for Endocrinology and Diabetes, National Center for Childhood Diabetes, Schneider Children’s Medical Center of Israel, Petach Tikva, Israel; ^3^ Azrieli Faculty of Medicine, Bar-Ilan University, Safed, Israel; ^4^ Department of Epidemiology and Preventive Medicine, School of Public Health, Faculty of Medicine, Tel Aviv University, Tel Aviv, Israel; ^5^ Felsenstein Medical Research Center, Tel Aviv University, Petach Tikva, Israel

**Keywords:** children, idiopathic short stature, germ-free mice, gut microbiota, gut metabolome, *methanobrevibacter*

## Abstract

**Objectives:**

To investigate the role of gut microbiota (GM) in pathogenesis of idiopathic short stature (ISS) by comparing GM of ISS children to their normal-height siblings.

**Methods:**

This case-control study, conducted at the Schneider Children’s Medical Center’s Institute for Endocrinology and Diabetes between 4/2018-11/2020, involved 30 pairs of healthy pre-pubertal siblings aged 3-10 years, each comprising one sibling with ISS and one with normal height. Outcome measures from fecal analysis of both siblings included GM composition analyzed by 16S rRNA sequencing, fecal metabolomics, and monitoring the growth of germ-free (GF) mice after fecal transplantation.

**Results:**

Fecal analysis of ISS children identified higher predicted levels of genes encoding enzymes for pyrimidine, purine, flavin, coenzyme B, and thiamine biosynthesis, lower levels of several amino acids, and a significantly higher prevalence of the phylum Euryarchaeota compared to their normal-height siblings (p<0.001). ISS children with higher levels of *Methanobrevibacter*, the dominant species in the archaeal gut community, were significantly shorter in stature than those with lower levels (p=0.022). Mice receiving fecal transplants from ISS children did not experience stunted growth, probably due to the eradication of *Methanobrevibacter* caused by exposure to oxygen during fecal collection.

**Discussion:**

Our findings suggest that different characteristics in the GM may explain variations in linear growth. The varying levels of *Methanobrevibacter* demonstrated within the ISS group reflect the multifactorial nature of ISS and the potential ability of the GM to partially explain growth variations. The targeting of specific microbiota could provide personalized therapies to improve growth in children with ISS.

## 1 Introduction

Short stature, defined as height < -2 standard deviations below the mean for age, sex, and population, is a common complaint in pediatric endocrinology (1, 2). It is considered idiopathic in the absence of systemic, endocrine, nutritional, or genetic abnormalities. Despite technological advances and extensive research, the underlying cause of idiopathic short stature (ISS) remains largely unknown. Studies in mice have shown that germ-free (GF) mice exhibit stunted growth and reduced weight gain on a standard breeding diet (3). Antibiotic treatment in GF mice colonized with gut microbiota (GM) of specific pathogen-free mice resulted in decreased serum insulin-like-growth-factor (IGF)-1 levels and inhibited bone formation, while supplementation of the antibiotic-treated mice with short-chain fatty acids, products of microbial metabolism, restored IGF-1 levels and bone mass to the levels of non-antibiotic-treated mice (3). Recent research in malnourished mice has shown that a strain of *Lactiplantibacillus plantarum* (Lp^WJL^) improved circulating levels and activity of IGF-1 and insulin by activating the NOD2 receptor in intestinal epithelial cells (4, 5). The role of nutrition and GM on linear growth in humans has mainly focused upon malnourished children in developing countries. Those studies found that a less diverse GM was associated with the severity of stunting (6), but such findings may be skewed due to differences in nutrition, sanitation, and healthcare accessibility. Indeed, genetic, environmental, and geographical factors are recognized as having an impact on the GM, prompting the consideration that members of the same family may have quite similar GM (7).

In the present study of the role of GM in the pathogenesis of ISS, we enrolled healthy pre-pubertal ISS children from an Israeli middle-class population and their normal-height siblings who served as controls. The study goals were to determine whether there is a difference in the GM composition between ISS children and their normal-height siblings and to investigate the relationship between GM and linear growth by transplanting feces from both groups into GF mice and monitoring their growth.

## 2 Methods

This case-control study was conducted at the Institute for Endocrinology and Diabetes at the Schneider Children’s Medical Center (SCMCI), Israel, from April 2018 through November 2020. It was approved by the Rabin Medical Center’s ethics review committee (study number 0238-17-RMC). All study participants’ parents signed a written informed consent form.

Patients: This study included 30 healthy pre-pubertal, short, and lean children (height ≤ 3^rd^ and body mass index (BMI) <10^th^ percentile for age and sex (2000 Centers of Disease Control [CDC] growth charts) from middle-class families referred to our clinic for growth assessment (one short child per family). In all ISS participants, a comprehensive medical work-up revealed no underlying systemic, hormonal, or skeletal pathologies as a cause of their ISS. Their general blood tests (complete blood count, renal and liver function tests, and a lipid profile performed at SCMCI’s central laboratory) fell within the normal range, and all were growth hormone (GH)‐sufficient with a peak stimulated GH levels ≥7.5 μg/L using the standard secretagogues of glucagon/clonidine/arginine.

The controls included their 30 healthy pre-pubertal siblings (one per participant) with normal percentiles of height (>25^th^), weight (25^th^-50^th^), and body mass index (BMI: 25^th^-75^th^).

The eligibility criteria for the ISS children and their siblings were as follows: Inclusion criteria: Born appropriate-for-gestational-age; aged 3-10 years; prepubertal - boys: testicular volume ≤3 mL; girls: breast Tanner stage 1; GH‐treatment naïve. Exclusion criteria: genetic syndromes, chromosomal abnormalities, known chronic diseases, gastrointestinal diseases, malabsorption syndromes, metabolic disorders, malignancy, overweight/obesity, use of medications affecting growth/appetite, and antibiotic use within 3 months prior to enrollment.

In Israel, genetic screening that includes a panel for short stature is carried out in accordance with the Ministry of Health guidelines. Genetic screening is performed only in children with a suspected genetic syndrome associated with short stature or a familial extreme short stature defined as more than 3 standard deviations below the mean. Since none of our participants fulfilled these criteria, they did not undergo genetic analysis such as G-banding and mutation screening of ISS causal genes.

### 2.1 Study demographic and nutritional questionnaires

Data on the demographics and family medical history of each participant and his/her sibling were collected upon study enrollment by means of individual questionnaires. Details of the perinatal history, neonatal feeding type (breast/formula), and use of antibiotics during the first year of life were also gathered. The anthropometric measurements of weight and height (by means of a Harpenden stadiometer, Holtain Ltd, Crosswell, United Kingdom) were taken on the same day, and BMI (weight [kg]/height [m]^2^) was calculated. Height and BMI were expressed as standard deviation scores (SDS) according to CDC recommendations (8). Pubertal staging was evaluated with the Tanner method (9). During the week preceding the fecal sample collection, the parents completed 2 questionnaires: the Child Eating Behavior Questionnaire (CEBQ) (10, 11) and a 3-day food diary (Tzameret 3 software. Israel Center for Disease Control and the Ministry of Health) (see **Supplementary Methods**) in order to assess the patients’ and siblings’ eating and food consumption patterns.

### 2.2 Fecal sample collection and processing

The parents used sterile marked test tubes and spoons to collect and store fecal samples from the study child and the matched sibling. They collected around 2 grams of fresh stools and stored the tubes in a home freezer at -20°C. The test tubes were delivered to the hospital on ice the following day and stored at -80°C until being shipped on dry ice to the Azrieli Faculty of Medicine at Bar-Ilan University in Safed, Israel (see **Supplementary Methods**).

### 2.3 Microbial 16S rRNA gene PCR amplification and sequencing

The PureLink Microbiome DNA Purification Kit was used to extract DNA from fecal samples after a 2-minute bead beating step (Invitrogen, Carlsbad, CA, USA). Bacterial 16S rRNA gene sequences were PCR-amplified with barcoded 515F-806R primers for the V4 hypervariable region of the gene as described in detail elsewhere (12, 13). Purified amplicons (AMPure, Beckman Coulter, Brea, California, USA) were quantified by PicoGreen assay (Molecular Probes, Eugene, OR). Pooled samples were purified with 2% E-Gel (Invitrogen, Carlsbad, CA) and sequenced on an Illumina MiSeq platform at the Azrieli Faculty of Medicine, Bar-Ilan University, Israel (13).

### 2.4 16S rRNA gene sequence analysis

Data analysis was performed using QIIME2 software (version 2020.2) (14). Sequence reads were de-multiplexed by per-sample barcodes and error-corrected by Divisive Amplicon Denoising Algorithm (DADA2) (15). The final feature sequences were aligned against the Greengenes database with 99% confidence for taxonomic annotation (16). To avoid contamination, the feature table was generated by filtering sequences of mitochondria, chloroplasts, S24-7, and *Candidatus*, as well as by filtering low abundance features (i.e., observed in fewer than 15% of the samples). The analysis was calculated on a rarefied table of >10400 reads per sample. Microbial richness, an alpha diversity parameter, was calculated by the Shannon Index and statistically compared between groups with a Kruskal-Wallis test. Beta diversity, which measures the compositional difference between groups, was characterized by weighted (quantitative) and unweighted (qualitative) UniFrac distances. To compare differences in gut bacterial communities between the sample groups, a permutational multivariate analysis of variance (PERMANOVA) test was performed, as implemented in QIIME2 with the default of 999 permutations for both weighted and unweighted UniFrac. Identification of differentially expressed bacteria was performed using Linear discriminant analysis Effect Size (LEfSe) (17). The logarithmic LDA score threshold was set to 2.0. A metagenome functional predictive analysis was carried out using phylogenetic investigation of communities by reconstruction of unobserved states (PICRUSt2) (18). Feature abundance was normalized by 16S rRNA gene copy number, identified, and compared to a phylogenetic reference tree using the Kyoto Encyclopedia of Genes and Genomes (KEGG) database to assign functional traits and abundances. A false discovery rate <0.1 was considered significant for multiple comparisons.

### 2.5 Metabolomics analysis

Twenty samples (10 from the ISS subgroup-S2 children [see below] and 10 from their siblings) were processed for targeted metabolomics of 66 metabolites (22 bile acids, 20 SCFAs, and 24 amino acids [AAs]) by Microbiota Metabolomics Technologies, Merck Israel Ltd., an affiliate of Merck KGaA, Darmstadt, Germany (**Supplementary Table 1**).

### 2.6 Fecal microbiota transplant to GF mice

The experiment protocol was approved by the Bar Ilan University Animal Studies Committee. Each donor fecal sample was individually suspended in 800 μl of sterile phosphate-buffered saline (PBS) and dissolved by vortex for 1 min. A fecal suspension from a single donor was administered by oral gavage to a pair of mice (150 μl per mouse) who were then co-housed in the same cage after the first transplant. Fecal samples from 12 ISS children (subgroup 2) and their matched siblings were transplanted to the 48 GF male mice by oral gavage (2 mice/sample). They received a second transplant from the same donor as the first transplant one week later. Mice were followed for 28 days post-transplant, with day 0 referring to the day of the first fecal transplantation; weight and fecal samples were collected weekly until the end of the experiment at 8 weeks of age (**Supplementary Methods**). The animals were euthanized and each mouse’s tibiae and humeri were removed, cleaned, and measured. Histological studies and epiphyseal growth plate (EGP) height measurements were performed as previously reported (19) (**Supplementary Methods**).

### 2.7 PCR analysis of a specific taxon (*Methanobrevibacter*)

Target taxa were chosen for finer quantification based upon preliminary 16S rRNA sequencing results. The PCR reaction, primer sequences, and PCR product sizes used in this study were described in a previous publication (20). The PCR primer specificities were validated *in silico* using the NCBI’s BLAST program (http://www.ncbi.nlm.nih.gov/BLAST).

### 2.8 Statistical analysis

Power analysis was not performed for the clinical study due to the lack of previous data on microbiota stability in healthy children. Unsupervised learning was conducted with Python version 3.5, and the Sklearn package was employed to identify patterns in the human microbiota data. SPSS software version 27 (Ref: IBM Corp. Released 2020. IBM SPSS Statistics for Windows, Version 27.0. Armonk, NY: IBM Corp) was used to analyze clinical and demographic data, with continuous data presented as mean ± standard deviation (SD: normal distribution) or median (interquartile range [IQR]: skewed distribution) and categorical variables presented as number and percentage. Comparisons between groups were made by using an independent samples t-test (normal distribution), Mann-Whitney U test (skewed distribution), or Pearson’s chi-squared test (for categorical variables). Spearman correlation analysis was conducted to find a correlation between *Methanobrevibacter* and height SDS. Significance was set at a *P* level of <.05.

## 3 Results

### 3.1 Participants

**Table 1** shows the clinical characteristics and auxological data at study enrollment of the 60 pre-pubertal healthy children (33 boys) who comprised the study cohort, including 30 short and lean children and their normal-height siblings. The participants’ ages ranged from 3.3 to 10 years, with the study group being significantly older than their siblings (*P* =.001). The study group also had a significantly lower weight SDS (*P* <.001), lower BMI-SDS (*P* = .03), and shorter height SDS (*P* <.001) than their siblings. Their perinatal characteristics were comparable, and no antibiotics were used in the first year of life for either group.

**Table 1:** Demographic and anthropometric characteristics of ISS children and their siblings according to the gut microbiome analysis.

Characteristic	All Participants			ISS Subgroup-S1			ISS Subgroup-S2			
	Subjects	Siblings	*P*	Subjects	Siblings	*P1*	Subjects	Siblings	*P2*	*P 1vs.2*
Number	30	30		14	14		16	16		
Males (%)	16 (53.3)	17 (56.7)	0.795	6 (42.9)	8 (57.1)	0.450	10 (62.5)	9 (56.3)	0.719	0.282
Age (y)	7.8 ± 2.0	5.9 ± 2.3	0.001	7.8 ± 1.8	6.2 ± 2.0	0.031	7.8 ± 2.2	5.6 ± 2.5	0.017	0.924
Height SDS	-2.1 (-2.22, -1.96)	-0.08 (-0.28, 0.59)	< 0.001	-2.00 (-2.01, -1.92)	0.27 (-0.25, 0.65)	< 0.001	-2.15 (-2.32, -2.00)	-0.15 (-0.39, 0.43)	< 0.001	0.022
Weight SDS	-1.85 ± 0.66	-0.04 ± 0.60	< 0.001	-1.78 ± 0.53	-0.07 ± 0.67	< 0.001	-1.91 ± 0.77	-0.02 ± 0.55	< 0.001	0.615
BMI SDS	-0.66 ± 0.95	-0.15 ± 0.81	0.03	-0.69 ± 0.76	-0.31 ± 1.01	0.265	-0.63 ± 1.11	-0.02 ± 0.59	0.064	0.858
GA (weeks)	39 (39,40)	39 (37,40)	0.465	38.5 (37,40)	38.5 (37,40)	0.804	39 (38,40)	39 (36.5,40)	0.590	0.418
C-section (%)	7 (23.3%)	6 (20.0%)	0.754	3 (21.4%)	2 (14.3%)	0.622	4 (25%)	4 (25%)	1	0.818
Birth weight (gr)	2970 ± 371	3137 ± 453	0.124	3032 ± 210	3220 ± 383	0.119	2916 ± 471	3063 ± 507	0.400	0.382
Breastfeeding (%)	24 (81)	24 (80)	1	12 (85.7)	12 (85.7)	1	12 (75)	12 (75)	1	0.464
MPHt-SDS	-0.42 ± 0.56			-0.39 ± 0.59			-0.46 ± 0.54			0.690
Peak GH	10.5(8.6,15)			10.5 (8.6,15.0)			10.2(8.3,14.2)			0.697

The clinical characteristics and auxological data at the time of enrollment. Data are presented as mean ± SD (normal distribution) or median (interquartile range, IQR) (skewed distribution), and as number (percentage) for categorical variables. P represents comparisons between all subjects (n = 30) and their siblings (n = 30). P1 represents comparisons between subjects from ISS subgroup-S1 and their siblings. P2 represents comparisons between subjects from ISS subgroup-S2 and their siblings. P1 vs. P2 represents comparisons between subjects from subgroup-S1 and subjects from ISS subgroup-S2.

ISS, idiopathic short stature; SDS, standard deviation score; BMI, body mass index; GA, gestational age; C-section, cesarean section; GH, growth hormone; MPHt, mid-parental height.

### 3.2 Eating behavior

Both ISS and the sibling groups consumed a balanced macronutrient diet (51% carbohydrate, 32% fat, and 16% protein) with the same nutrient intake. Most of the participants had appropriate daily micronutrient intake, but calcium, iron, and vitamin C consumption levels were lower than recommended for both groups. Participants in the ISS group scored lower in food enjoyment (*P* = .019) and food responsiveness (*P* = .004) and higher in slowness in eating and satiety responsiveness (*P* <.001 for both), compared to their siblings (**Tables 2A, B**).

### 3.3 Microbiota analysis

**Figure 1:**
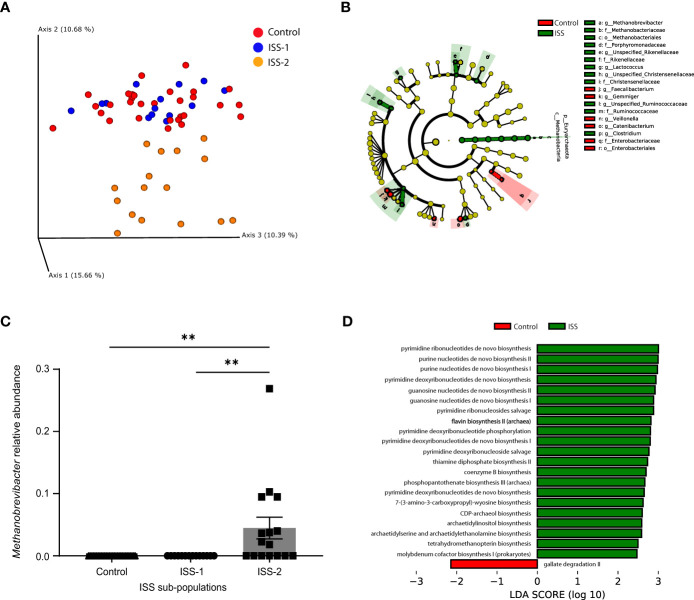
Children with ISS display distinct microbiome characteristics compared to their normal-height siblings. **(A)** Principal coordinates analysis (PCoA) based upon the Unweighted UniFrac matrices of fecal samples collected from children with ISS and their normal-weight and -height siblings. Each dot represents an individual, colored by the experimental group. **(B)** Taxonomic cladogram illustrating the most differentially enriched taxa using linear discriminant analysis Effect Size (LEfSe) analysis. (LDA >2). Groups are identified by the different colors. **(C)** Relative abundance of *Methanobrevibacter* within the 3 groups (controls and the 2 subpopulations of the ISS group, (**P <.001). **(D)** Computed Linear Discriminant Analysis (LDA) scores for the differentially enriched microbial metabolic pathways using PICRUSt2 analysis. (LDA >2), C-control, S-ISS.

Alpha diversity was similar between children with ISS and their siblings (data not shown). Beta diversity, calculated by using weighted (quantitative) and unweighted (qualitative) UniFrac, revealed significant differences in the distance metrics between gut bacterial communities of the 2 groups *P* <.01). The sibling group’s samples clustered together, indicating a high degree of similarity, whereas the ISS group’s samples had a higher degree of dissimilarity, with an internal division into 2 subpopulations, one more similar to the siblings (subgroup-S1), and the other entirely dissimilar (subgroup-S2) (**Figure 1A**).

The main taxa at the phylum and genus levels for the ISS children and their siblings were similar (Firmicutes, Actinobacteria, Bacteroidetes, Verrucomicrobia, and Proteobacteria), with differences in the abundance of the phylum Euryarchaeota (i.e., over-represented in the ISS group as confirmed by LefSe analysis). This significant difference was confirmed across all Euryarchaeota taxonomic levels (Methanobacteria, Methanobacteriales, Methanobacteriaceae, and *Methanobrevibacter)* (**Figure 1B**). Furthermore, the ISS subgroup-S2 had significantly higher levels of the genus *Methanobrevibacter* compared to the rest of the study population (**Figure 1C**). *Post hoc* analysis comparing the two ISS sub-groups was conducted based on these differences (**Figure 1A**). PICRUSt2, which predicts the functional/metabolic potential of a bacterial community based upon marker gene sequencing profiles, showed that the ISS group had significantly higher levels of genes encoding enzymes involved in *de novo* biosynthesis of pyrimidines and purines, flavin, coenzyme B, and thiamine (**Figure 1D**).

#### 3.3.1 Comparison of the clinical measures of the 2 ISS subgroups classified according to GM clusters

Based on the GM findings, we divided the ISS group into 2 subgroups. The only difference in anthropometric measurements between the 2 ISS subgroups was that subgroup-S2 had a significantly lower height SDS (*P* = .022) than subgroup-S1 (**Table 1**). Additionally, a significant, moderate, negative correlation was found between the level of *Methanobrevibacter* and height SDS in the ISS group (rs=-0.371; p=0.044). There were no differences in their nutritional intake, and dietary eating patterns (**Table 2**). Laboratory tests revealed that the 2 subgroups had a similar hormonal profile (**Table 3**). However, subgroup-S2 had lower levels of alkaline phosphatase (*P* <.001), iron (*P* <.001), ferritin (*P* = .038), and their hemoglobin levels were slightly lower (*P* = .058) (**Tables 1**, **2A, B**).

**Table 2:** Food consumption and dietary eating patterns of the ISS children and their siblings according to the gut microbiome analysis.

Characteristic	All Participants			ISS Subgroup-S1			ISS Subgroup-S2			
	Subjects	Siblings	*P*	Subjects	Siblings	*P1*	Subjects	Siblings	*P2*	*P 1vs.2*
A. Food consumption										
Number	28	27		13	12		15	15		
Number of meals/day	4.7 (4.7, 5.0)	5.0 (4.7, 5.3)	0.229	4.7 (4.7, 5.0)	5.0 (4.7, 5.0)	0.179	5.0 (4.3, 5.1)	5.0 (4.6, 5.7)	0.561	0.565
Energy/wt (Kcal/kg/day)	75.3 ± 15.5	70.4 ± 23.3	0.357	73.0 ± 16.9	66.0 ± 16.7	0.310	77.3 ± 14.5	73.9 ± 27.5	0.670	0.468
Protein/wt (gr/kg/day)	3.2 ± 0.8	2.9 ± 1.2	0.312	3.1 ± 0.9	2.7 ± 0.8	0.191	3.2 ± 0.8	3.0 ± 1.4	0.753	0.946
% Kcals from Proteins	16.8 ± 2.7	16.1 ± 2.3	0.303	17.2 ± 2.6	16.3 ± 2.2	0.327	16.5 ± 2.9	16.0 ± 2.5	0.628	0.475
Dairy/total proteins (%)	20.7 ± 9.7	20.9 ± 10.9	0.932	19.1 ± 8.8	18.0 ± 10.2	0.772	22.1 ± 10.6	23.3 ± 11.3	0.762	0.427
Meat/total proteins (%)	41.2 ± 15.8	38.7 ± 14.4	0.547	43.6 ± 13.5	41.1 ± 11.8	0.629	39.1 ± 17.7	36.8 ± 16.4	0.713	0.460
Fat/wt (gr/kg/day)	2.5 ± 0.6	2.2 ± 0.8	0.161	2.4 ± 0.7	2.0 ± 0.4	0.085	2.5 ± 0.6	2.4 ± 1.0	0.597	0.679
% Kcals from fats	29.9 ± 4.4	28.7 ± 3.8	0.280	30.1 ± 4.6	28.2 ± 3.1	0.241	29.8 ± 4.4	29.1 ± 4.4	0.686	0.856
Carbs/wt (gr/kg/day)	9.7 ± 2.2	9.4 ± 3.0	0.660	9.4 ± 2.2	9.0 ± 2.6	0.705	10.1 ± 2.2	9.8 ± 3.4	0.779	0.442
% Kcals from carbs	51.8 ± 5.9	53.8 ± 4.3	0.166	51.7 ± 5.9	54.3 ± 4.5	0.238	52.0 ± 6.1	53.4 ± 4.3	0.449	0.914
Zinc (% RDA)	100.3 (82.6, 130.4)	133.6 (98.6, 186.8)	0.080	85.9 (75.4,122.1)	128.4 (76.1, 175.1)	0.384	110.0 (97.2,133.4)	142.4 (101.7,200.3)	0.152	0.076
Iron (% RDA)	83.0 (64.9, 115.4)	74.1 (61.9, 116.0)	0.692	80.6 (66.4,121.0)	79.0 (53.4, 128.8)	0.624	83.2 (62.1,103.2)	74.1 (71.3,94.7)	0.604	0.945
Calcium (% RDA)	50.3 ± 24.6	53.3 ± 23.1	0.647	53.1 ± 30.9	46.6 ± 21.6	0.549	47.9 ± 18.5	58.7 ± 23.6	0.175	0.589
B. Dietary eating patterns										
Number	29	29		14	14		15	15		
**Food responsiveness**	1.4 (1.2, 1.7)	1.6 (1.4, 2.5)	**0.004**	1.3 (1.2, 1.8)	2.0 (1.4, 3.0)	**0.039**	1.4 (1.2, 1.6)	1.5 (1.4, 2.0)	**0.045**	0.747
**Enjoyment of food**	3.0 (2.3, 3.5)	3.5 (2.9, 4.0)	**0.019**	2.8 (2.3, 3.6)	3.4 (3.0, 4.0)	**0.024**	3.0 (2.0, 3.5)	3.5 (2.8, 4.0)	0.300	0.893
Emotional overeating	1.3 (1.0, 2.0)	1.5 (1.0, 2.3)	0.464	1.3 (1.0, 2.0)	1.3 (1.0, 2.5)	0.571	1.5 (1.0, 2.0)	1.8 (1.0, 2.0)	0.797	0.687
Desire to drink	1.7 (1.3, 2.8)	2.0 (1.5, 3.5)	0.214	1.8 (1.3, 2.8)	2.7 (1.7, 3.7)	0.164	1.7 (1.3, 3.0)	2.0 (1.3, 3.0)	0.748	0.767
**Satiety responsiveness**	3.8 (3.4, 4.2)	2.6 (2.4, 3.6)	**<0.001**	3.7 (3.4, 4.3)	2.6 (2.4, 3.3)	**0.001**	4.0 (3.4, 4.2)	3.2 (2.6, 3.8)	**0.019**	0.715
**Slowness of eating**	3.3 (2.9, 4.0)	2.5 (2.0, 3.0)	**<0.001**	3.1 (2.9, 3.6)	2.3 (2.0, 3.0)	**0.011**	3.5 (2.8, 4.3)	2.5 (2.3, 3.0)	**0.002**	0.451
Emotional under eating	2.8 (2.0, 3.3)	2.5 (2.0, 3.4)	0.938	2.9 (1.9, 3.3)	2.8 (1.9, 3.6)	0.946	2.8 (2.3, 3.3)	2.5 (2.0, 3.0)	0.748	0.999
Food fussiness	3.3 (2.3, 3.8)	3.0 (2.2, 3.8)	0.846	3.4 (2.3, 4.0)	3.7 (2.8, 4.0)	0.734	3.3 (2.2, 3.7)	2.3 (2.2, 3.7)	0.436	0.477

P1 represents comparisons between subjects from ISS subgroup-S1 and their siblings. P2 represents comparisons between subjects from ISS subgroup-S2 and their siblings. P1 vs. P2 represents comparisons between subjects from ISS subgroup-S1 and subjects from ISS subgroup-S2. **A:** Food consumption by the ISS children and their siblings subdivided according to the gut microbiota analysis **B**: Dietary intake patterns by the ISS children and their siblings subdivided according to the gut microbiota analysis.

Bold indicates significant.

ISS, idiopathic short stature; RDA- recommended dietary allowance.

**Table 3:** Blood metabolic and hormonal profiles.

Characteristic	ISS Subgroup 1	ISS Subgroup 2	*P*
Number	14	16	
Peak GH (ng/ml)	10.5 (8.6, 15.0)	10.2 (8.3, 14.2)	0.697
IGF1 (ng/ml)	118.3 ± 26.5	110.8 ± 42.8	0.576
TSH (mIU/ml)	1.9 ± 0.4	2.4 ± 0.6	**0.042**
FT4 (pmol/L)	15.6 ± 1.3	14.3 ± 1.9	**0.035**
Hb (gr/dl)	12.1 (11.8, 12.7)	11.8 (11.5, 12.2)	**0.058**
Iron (mcg/dL)	74.5 (68.5, 79.5)	48.5 (38.3, 64.0)	**<0.001**
Ferritin (ng/ml)	33.5 (25.0, 43.9)	25.9 (19.2, 32.9)	**0.038**
Albumin (gr/dL)	4.4 ± 0.3	4.3 ± 0.4	0.735
Alk-phos (IU/L)	215.7 ± 33.2	158.9 ± 20.3	**<0.001**
Total cholesterol (mg/dL)	158.0 (140.8, 163.4)	153.1 (128.2, 162.8)	0.525
HDL cholesterol (mg/dL)	57.4 ± 8.6	53.6 ± 12.8	0.356
LDL cholesterol (mg/dL)	82.1 ± 13.7	82.1 ± 14.8	0.988
TG (mg/dL)	62.0 ± 19.0	59.9 ± 22.6	0.784

Bold indicates significant.

GH, growth hormone; IGF1- Insulin like growth factor 1; TSH – thyroid stimulating hormone; FT4 – thyroxine; Hb, hemoglobin; Alk-phos, alkaline phosphatase.

### 3.4 Fecal metabolic analysis

**Figure 2:**
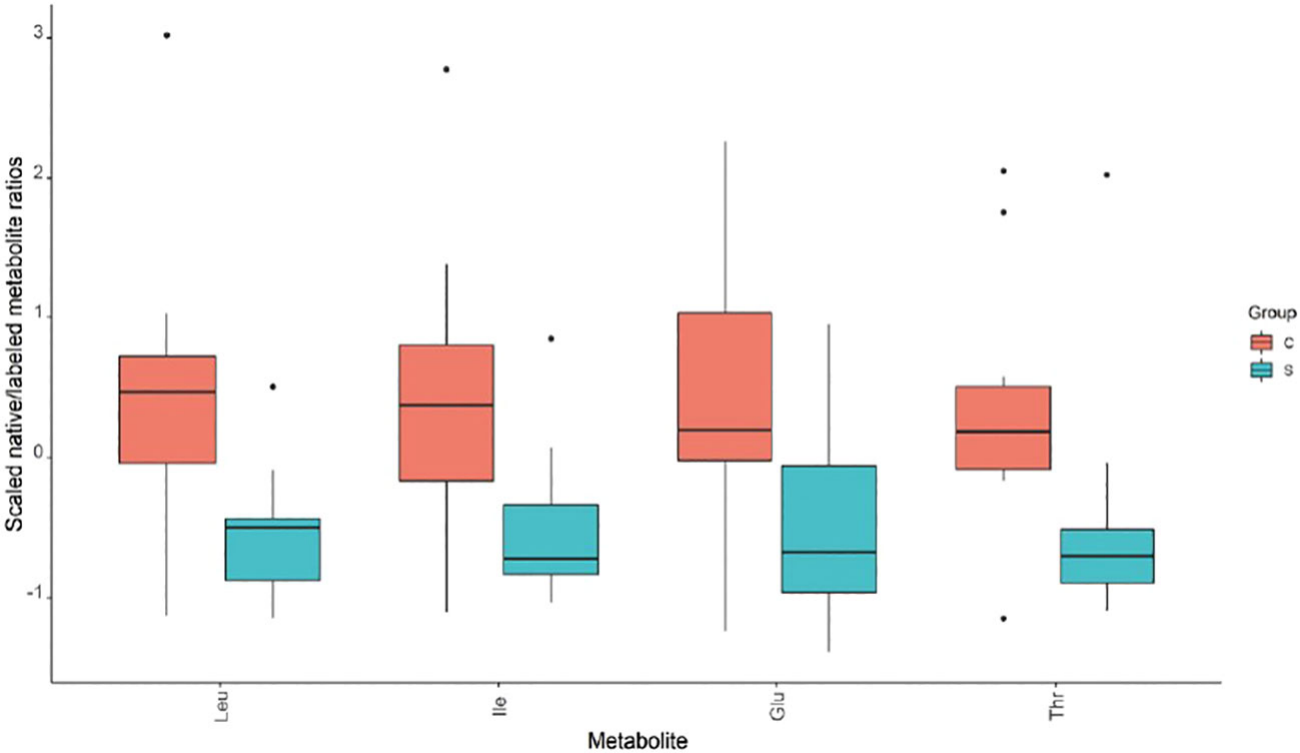
Metabolic analysis revealed significant differences in amino acid levels between ISS subgroup S2 and its controls.

The metabolic analysis of 66 metabolites did not reveal any significant difference between the 2 study groups, except for 4 amino acids (AAs) (Leucine, Isoleucine, Glutamic acid, and Threonine) that were significantly reduced in children from subgroup-S2 (Wilcoxon *P* <.05) (**Figure 2**, **Supplementary Table 1**).

### 3.5 Response of GF mice to fecal microbiota transplantation

**Figure 3:**
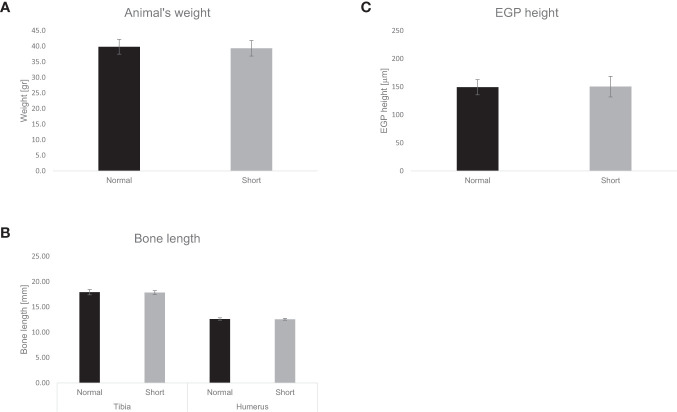
Effect of fecal transplantation on murine weight, bone length and EGP height. The effect of fecal transplantation from children with ISS (short) compared to their normal height siblings (normal) on animals’ **(A)** weight. **(B)** Tibia and humerus length, **(C)** EGP height. Mean± SD values are shown.

Mice transplanted with feces from ISS subgroup-S2 and from their siblings showed no differences in weight, weight gain, or food consumption during the 28-day follow-up after transplantation. No significant differences were observed in the length of humeri or tibia, in the height of the EGP, or in the organization of chondrocytes (21) (**Figure 3**).

### 3.6 PCR analysis of *Methanobrevibacter*

To investigate the lack of stunted growth in mice that received fecal transplants from children with ISS, we conducted PCR to determine the presence of *Methanobrevibacter* in the fecal samples of the transplanted mice. *Methanobrevibacter* was present only in samples taken from the children in subgroup-S2 and not from the transplanted GF mice.

## 4 Discussion

The results of this analysis of the GM of children with ISS and their normal-height siblings revealed that the phylum Euryarchaeota was present exclusively in the ISS group. Within the ISS group, the GM analysis identified two subgroups, with *Methanobrevibacter*, the predominant species in the ISS group’s archaeal gut community, found solely in subgroup 2. *Post hoc* analysis comparing the two ISS subgroups disclosed that children in subgroup 2 were shorter, and their height SDS exhibited a significant negative correlation with the abundance of *Methanobrevibacter*. This observation of diversity within the ISS group is innovative and implies a potential role of GM in understanding variations in the underlying causes of ISS.

ISS is a complex term that encompasses underlying mechanisms that are still unidentified. The GM and growth have recently been connected, with microbial diversity correlated with stunting severity (6). Our findings suggest that the composition of GM may play a role in explaining disparities in linear growth in some of the ISS children participating in this study, namely children with higher *Methanobrevibacter* levels (ISS-subgroup 2). However, in the ISS-subgroup with GM similar to that of their normal height siblings (ISS-subgroup 1) the underlying causes of ISS may be attributed to a wide range of other as-yet-unidentified variables.

In contrast to Li et al.’s recent study (22), which reported variations in GM composition and metabolite levels between children with ISS and the general population in eastern China, our analysis revealed no difference in microbial richness between children with ISS and their siblings. However, we did observe 3 notable variations, including the presence of *Methanobrevibacter*, which not only distinguished the short children from their normal-height siblings but also delineated 2 distinct subclasses within the ISS group. We also noted increased predicted levels of metabolic enzymes and decreased levels of specific AAs in those children. The differences between our findings and those of Li et al. could be attributed to differences in study design. While the former authors compared children with ISS to normal-height children from the general population, we conducted a comparison of each ISS child to his/her normal-height sibling, thereby isolating differences related to short stature rather than genetics, home environment, dietary habits, or demographics.

The only clinical difference between the 2 subgroups of ISS was the significantly shorter stature of the subgroup displaying elevated levels of *Methanobrevibacter.* Serum levels of Alkaline Phosphatase, a potential marker of bone elongation and osteoblast differentiation, were also significantly low in ISS Subgroup 2. This finding suggests that *Methanobrevibacter*, possibly through specific metabolites, may have an adverse impact on chondrocytes and osteoblasts, consequently impeding bone elongation. Nonetheless, in our study cohort, the causal relationship between *Methanobrevibacter* and ISS remains unidentified. Food consumption and eating behavior were also similar in the two subgroups. These findings are in opposition to those of previous studies that had suggested that the presence of *Methanobrevibacter* is influenced by dietary habits as evidenced by elevated levels of *Methanobrevibacter* in the fecal samples of malnourished and anorexic patients (23). Previous research has demonstrated that the diverse roles of certain *Methanobrevibacter* species are often associated with complex sugar degradation. Additionally, several studies demonstrated that *Methanobrevibacter* can increase short-chain fatty acid (SCFA) production, either directly or through association with other bacterial strains in gut (24). Contrarily, our metabolomics analysis failed to identify any significant differences between the ISS children and their siblings. It is plausible that *Methanobrevibacter* might behave differently in healthy, well-nourished individuals that possess stable microbiota. Although our analysis did not identify significant differences between the groups, these assumptions are based on existing literature. In this study we focused on a panel of 66 bacterially associated metabolites. We found no evidence of altered complex sugar degradation, nor did PiCRUSt predictive analysis reveal any differentially abundant metabolites/pathways associated with *Methanobrevibacter*. More research would be needed to more deeply understand the role of this taxon in ISS.

Our bioinformatics analysis suggested a possible increase in genes encoding enzymes involved in the *de novo* biosynthesis of pyrimidines and purines, as well as flavin, thiamine, and other similar compounds in the ISS group (**Figure 1D**). Pyrimidines and purines are essential building blocks of DNA and RNA, and also play important roles in various metabolic and signaling processes. Flavin mononucleotide and flavin adenine dinucleotide are crucial active groups in most flavoproteins/flavo-coenzymes, which are involved in numerous vital physiological functions (25). However, it remains unclear what clinical significance an increase in fecal levels of these compounds would have in children with ISS.

Furthermore, when compared to their siblings, the ISS children had lower levels of the AAs leucine, isoleucine, glutamic acid, and threonine in their feces. Colonic bacteria use these AAs to produce a complex mixture of metabolic end-products, including SCFAs, which affects metabolism and growth by changing hormone levels, such as leptin, ghrelin, glucagon-like peptide 1, and peptide YY, as well as by increasing IGF-1 in serum, liver, and bone (3). Leucine, in particular, is a strong inducer of the mammalian target of rapamycin complex 1 (mTORC1), a protein complex that functions as a nutrient sensor as well as a regulator of protein synthesis (26). Furthermore, leucine is required for IGFBP-1 de-phosphorylation: without it, IGFBP-1 has significantly increased IGF-1 affinity, stabilizing the IGF-1-IGFBP-1-ALS complex and inhibiting IGF-1-stimulated cell growth (27). Although we did not find lower levels of SCFA in our ISS group, an association of lower levels of these AAs with slower linear growth cannot be ruled out.

### 4.1 Strength and limitations

The strength of the study lies in its design – it was conducted in a single tertiary care center with a uniform standard of care. Furthermore, the control of each ISS child with his/her normal–height sibling enabled reduction of the bias of confounding factors such as genetics, living environment, and dietary habits. The main limitation of our study was the fecal transplantation outcome. The current study sought to investigate the relationship between GM and linear growth by using a fecal transplant from the ISS subgroup-S2 and their healthy siblings into GF mice. We were disappointed to not have observed any differences between the 2 GF mouse groups, but rather similar weight gain, linear growth, and EGP height. It is possible that *Methanobrevibacter*, an anaerobic archaeon, may have perished due to exposure to oxygen during fecal collection and/or transplantation. Indeed, based on our PCR analysis, *Methanobrevibacter* was detected, in the fecal samples of children from ISS subgroup-S2, yet it was absent in all of the mouse samples that received these fecal transplants. The absence of *Methanobrevibacter* in the transplanted feces may explain why the mouse recipients of fecal transplants from ISS subgroup-S2 children did not exhibit stunted growth. Our findings underscore a significant challenge in microbiota research, as the utilization of feces as a proxy for GM is common, but the collection of fresh fecal samples might result in the demise of anaerobic bacteria, therefore we found that it is important to present the data of the transplantation. Further limitations of this study include our use of a 16S rRNA gene analysis instead of a shotgun approach and the constraints of our targeted metabolomics analysis.

## 5 Conclusions

Aside from the differences in GM between ISS children and their normal-height siblings, our study findings revealed that the ISS group itself is diverse, with varying levels of *Methanobrevibacter*. These findings highlight the potential role of the GM in explaining variations in linear growth among healthy children and could serve as a starting point for personalized therapies targeting the GM to improve growth in children with ISS.

## Data availability statement

The data presented in the study are deposited in the EBI repository, accession numbers: PRJEB72222 and ERP157006.

## Ethics statement

The studies involving humans were approved by Rabin Medical Center’s ethics review committee (study number 0238-17-RMC). The studies were conducted in accordance with the local legislation and institutional requirements. Consent to participate in this study was obtained from the participants legal guardians/next of kin. The animal study was approved by Bar Ilan University Animal Studies Committee. The study was conducted in accordance with the local legislation and institutional requirements.

## Author contributions

LL: Conceptualization, Data curation, Formal analysis, Supervision, Validation, Writing – original draft, Writing – review & editing. AE: Writing – review & editing, Investigation, Methodology. LM: Methodology, Writing – review & editing, Data curation. MY-G: Writing – review & editing, Formal analysis. MB: Writing – review & editing, Methodology, Data curation. BS: Methodology, Writing – review & editing, Data curation. MN: Writing – review & editing, Formal analysis, Funding acquisition. MP: Funding acquisition, Writing – review & editing. ST: Writing – review & editing, Data curation, Formal analysis. OK: Formal analysis, Writing – review & editing, Supervision. GG: Formal analysis, Supervision, Writing – review & editing, Conceptualization, Data curation, Investigation, Validation, Writing – original draft.
