# Supplementary material for: Children with idiopathic short stature have significantly different gut microbiota than their normal height siblings: a case-control study
Source: Front Endocrinol (Lausanne). 2024 Feb 23;15:1343337. doi: 10.3389/fendo.2024.1343337 (PMC10920232; doi:10.3389/fendo.2024.1343337)
Supplement: Supplementary file 1 [file Table_1.docx]

Supplementary Material

**Supplementary table 1**

**1A: Metabolite list for bile acids (BAs)**

*#* **Metabolite Abbreviation BA class HMDB# Ion*^a^* Normalized to**

| *1* | Tauroursodeoxycholic acid | TUDCA | Taurine-conjugated | HMDB0000874 | [M-H] | TCA-*d*_4_ |
| --- | --- | --- | --- | --- | --- | --- |
| *2* | Taurocholic acid | TCA | Taurine-conjugated | HMDB0000036 | [M-H] | TCA-*d*_4_ |
| *3* | Glycoursodeoxycholic acid | GUDCA | Glycine-conjugated | HMDB0000708 | [M-H] | TCA-*d*_4_ |
| *4*  *5*  *6*  *7*  *8*  *9*  *10*  *11*  *12*  *13*  *14*  *15*  *16*  *17*  *18* | Glycocholic acid | GCA | Glycine-conjugated | HMDB0000138 | [M-H] | TDCA-*d*_4_ |
|  | Taurochenodesoxycholic acid | TCDCA | Taurine-conjugated | HMDB0000951 | [M-H] | TDCA-*d*_4_ |
|  | Taurodeoxycholic acid | TDCA | Taurine-conjugated | HMDB0000896 | [M-H] | TDCA-*d*_4_ |
|  | Sulfolithocholylglycine acid | sGLCA | Glycine-conjugated & sulfated | HMDB0002639 | [M-H] | CA-*d*_5_ |
|  | Cholic acid | CA | BA | HMDB0000619 | [M+FA-H] | CA-*d*_5_ |
|  | Ursodeoxycholic acid | UDCA | BA | HMDB0000946 | [M+FA-H] | CA-*d*_5_ |
|  | Hyodeoxycholic acid | HDCA | BA | HMDB0000733 | [M-H] | CA-*d*_5_ |
|  | Glycochenodeoxycholic acid | GCDCA | Glycine-conjugated | HMDB0000637 | [M-H] | CA-*d*_5_ |
|  | Glycodeoxycholic acid | GDCA | Glycine-conjugated | HMDB0000631 | [M-H] | CA-*d*_5_ |
|  | Chenodeoxycholic acid | CDCA | BA | HMDB0000518 | [M+FA-H] | CA-*d*_5_ |
|  | Deoxycholic acid | DCA | BA | HMDB0000626 | [M-H] | CA-*d*_5_ |
|  | Isolithocholic acid | IsoLCA | BA | HMDB0000717 | [M-H] | LCA-*d*_5_ |
|  | Lithocholic acid | LCA | BA | HMDB0000761 | [M-H] | LCA-*d*_5_ |
|  | Deoxycholic acid 3-sulfate | sDCA | Sulfated | HMDB0002504 | [M-H] | CA-*d*_5_ |
|  | Glycolithocholic acid | GLCA | Glycine-conjugated | HMDB0000698 | [M-H] | LCA-*d*_5_ |
| *19* | Hyocholic acid | HCA | BA | HMDB0000760 | [M+FA-H] | TDCA-*d*_4_ |
| *20*  *21*  *22* | 3-Oxocholic acid | 3OCA | BA | HMDB0000502 | [M-H] | CA-*d*_5_ |
|  | Chenodeoxycholic acid 3-sulfate | sCDCA | Sulfated | HMDB0002586 | [M-H] | CA-*d*_5_ |
|  | Lithocholic acid 3-sulfate | sLCA | Sulfated | HMDB0000907 | [M-H] | CA-*d*_5_ |

*^a^*Ion used in MS1 analysis.

**1B: Metabolite list for short-chain fatty acids (SCFAs)**

*#* **Metabolite Abbreviation SCFA class HMDB# Ion Normalized to**

| *1* | Acetic acid | Ace | SCFA |  | HMDB0000042 | [M-H] | Acetic acid-^13^C_2_ |
| --- | --- | --- | --- | --- | --- | --- | --- |
| *2*  *3* | Propionic acid | Prop | SCFA |  | HMDB0000237 | [M-H] | Propionic acid-^13^C_3_ |
|  | Butyric acid | But | SCFA |  | HMDB0000039 | [M-H] | Butyric acid-^13^C_4_ |
| *4* | Isobutyric acid | iBut | SCFA |  | HMDB0001873 | [M-H] | Butyric acid-^13^C_4_ |
| *5*  *6*  *7*  *8*  *9*  *10*  *11*  *12*  *13*  *14*  *15*  *16*  *17*  *18*  *19*  *20* | 2-Methylbutyric acid | 2MeBut | SCFA |  | HMDB0002176 | [M-H] | Butyric acid-^13^C_4_ |
|  | Valeric acid | Val | SCFA |  | HMDB0000892 | [M-H] | Butyric acid-^13^C_4_ |
|  | Isovaleric acid | iVal | SCFA |  | HMDB0000718 | [M-H] | Butyric acid-^13^C_4_ |
|  | Isocaproic acid | iCap | SCFA |  | HMDB0000689 | [M-H] | Butyric acid-^13^C_4_ |
|  | Caproic acid | Cap | SCFA |  | HMDB0000535 | [M-H] | Butyric acid-^13^C_4_ |
|  | D-Glyceric acid | Glyceric | SCFA-OH |  | HMDB0000139 | [M-H] | Acetic acid-^13^C_2_ |
|  | Glycolic acid | Glyco | SCFA-OH |  | HMDB0000115 | [M-H] | Acetic acid-^13^C_2_ |
|  | 3-Hydroxypropionic acid | 3HPro | SCFA-OH |  | HMDB0000700 | [M-H] | Acetic acid-^13^C_2_ |
|  | L-Lactic acid | L-Lact | SCFA-OH |  | HMDB0000190 | [M-H] | Acetic acid-^13^C_2_ |
|  | 3-Hydroxybutyric acid | 3HBut | SCFA-OH |  | HMDB0000011 | [M-H] | Acetic acid-^13^C_2_ |
|  | 2-Hydroxybutyric acid | 2HBut | SCFA-OH |  | HMDB0000008 | [M-H] | Propionic acid-^13^C_3_ |
|  | D/L-Malic acid | Malic | Dicarboxyl |  | HMDB0031518/HMDB0000156 | [M-H] | Butyric acid-^13^C_4_ |
|  | Malonic acid | Malon | Dicarboxyl |  | HMDB0000691 | [M-H] | Butyric acid-^13^C_4_ |
|  | Succinic acid | Succin | Dicarboxyl |  | HMDB0000254 | [M-H] | Butyric acid-^13^C_4_ |
|  | Glutaric acid | Glutar | Dicarboxyl |  | HMDB0000661 | [M-H] | Butyric acid-^13^C_4_ |
|  | Fumaric acid | Fumar | Dicarboxyl |  | HMDB0000134 | [M-H] | Butyric acid-^13^C_4_ |

**1C: Metabolite list amino acids (AAs)**

*#* **Metabolite Abbreviation AA class HMDB# Ion Normalized to**

| *1* | Pyroglutamic acid | Glp | α amino acids and derivatives | HMDB0000267 | [M+H] | Phenyl-^13^C_6_-alanine |
| --- | --- | --- | --- | --- | --- | --- |
| *2*  *3* | Phenylalanine | Phe | Phenylalanine and derivatives | HMDB0000159 | [M+H] | Phenyl-^13^C_6_-alanine |
|  | Tryptophan | Trp | Indolyl carboxylic acids and derivatives | HMDB0000929 | [M+H] | Phenyl-^13^C_6_-alanine |
| *4* | Leucine | Leu | Leucine and derivatives | HMDB0000687 | [M+H] | Leucine-^13^C_6_,^15^N |
| *5*  *6*  *7*  *8*  *9*  *10*  *11*  *12*  *13*  *14*  *15*  *16*  *17*  *18*  *19*  *20* | Isoleucine | Ile | Isoleucine and derivatives | HMDB0000172 | [M+H] | Isoleucine-^13^C_6_,^15^N |
|  | Creatinine | Cr | α amino acids and derivatives | HMDB0000562 | [M+H] | Methionine-^13^C_5_,^15^N |
|  | Methionine | Met | Methionine and derivatives | HMDB0000696 | [M+H] | Methionine-^13^C_5_,^15^N |
|  | Tyrosine | Tyr | Tyrosine and derivatives | HMDB0000158 | [M+H] | Tyrosine-(phenyl-^13^C_6_) |
|  | Proline | Pro | Proline and derivatives | HMDB0000162 | [M+H] | Proline-^13^C_5_ |
|  | Valine | Val | Valine and derivatives | HMDB0000883 | [M+H] | Valine-^13^C_5_ |
|  | α-Aminobutyric acid | Abu | L-α-amino acids | HMDB0000452 | [M+H] | Proline-^13^C_5_ |
|  | Glutamic Acid | Glu | Glutamic acid and derivatives | HMDB0000148 | [M+H] | Glutamic acid-^13^C_5_ |
|  | Threonine | Thr | L-α-amino acids | HMDB0000167 | [M+H] | Threonine-^13^C_4_ |
|  | Glutamine | Gln | L-α-amino acids | HMDB0000641 | [M+H] | Cystine-^13^C_6_,^15^N_2_ |
|  | Serine | Ser | Serine and derivatives | HMDB0000187 | [M+H] | Cystine-^13^C_6_,^15^N_2_ |
|  | 5-Aminopentanoic acid | 5Ava | Δ amino acids and derivatives | HMDB0003355 | [M+H] | Cystine-^13^C_6_,^15^N_2_ |
|  | Citrulline | Cit | L-α-amino acids | HMDB0000904 | [M+H] | Cystine-^13^C_6_,^15^N_2_ |
|  | Cystine | Cys2 | L-cysteine-S-conjugates | HMDB0000192 | [M+H] | Cystine-^13^C_6_,^15^N_2_ |
|  | Asymmetric dimethylarginine | ADMA | Arginine and derivatives | HMDB0001539 | [M+H] | Histidine-^13^C_6_ |
|  | Histidine | His | Histidine and derivatives | HMDB0000177 | [M+H] | Histidine-^13^C_6_ |
| *21* | 3-Methylhistidine | 3MH | Histidine and derivatives | HMDB0000479 | [M+H] | Histidine-^13^C_6_ |
| *22*  *23*  *24* | Lysine | Lys | L-α-amino acids | HMDB0000182 | [M+H] | Lysine-^13^C_6_ |
|  | Ornithine | Orn | L-α-amino acids | HMDB0000214 | [M+H] | Lysine-^13^C_6_ |
|  | Arginine | Arg | L-α-amino acids | HMDB0000517 | [M+H] | Arginine-^13^C_6_ |
