# Supplementary material for: Children with idiopathic short stature have significantly different gut microbiota than their normal height siblings: a case-control study
Source: Front Endocrinol (Lausanne). 2024 Feb 23;15:1343337. doi: 10.3389/fendo.2024.1343337 (PMC10920232; doi:10.3389/fendo.2024.1343337)
Supplement: Supplementary file 2 [file DataSheet_1.docx]

Supplementary Material

# Supplementary Methods

1. **Study questionnaires (Nutritional Data):** The parents completed 2 additional questionnaires to assess the dietary intake patterns and the consumption of groups of foods by the patients and the siblings (controls): 1) The Child Eating Behavior Questionnaire (CEBQ) 10, a validated tool consisting of a 35-item parent report designed to assess a range of eating styles in children. Parents rate the frequency of their child’s behaviors on a 5-point Likert scale (1-never, 2-rarely, 3-sometimes, 4-often, and 5-always). The CEBQ has good internal consistency, test-retest reliability, and stability over time, and it has been shown to be related to food intake in behavioral tests 10 Validation of the Hebrew version of the CEBQ was described elsewhere 11. 2) A 3-day food diary during the week preceding the fecal sample collection. After undergoing brief training by a dietician, the parents were asked to record all food and beverages consumption, including portion sizes, consumed over a 3-day period (2 weekdays and 1 weekend). Dietary intake, including the analysis of the amount of protein, carbohydrate, fat composition as well as vegetarian vs. animal protein consumption, was analyzed using Tzameret 3 software (Tzameret 3 software. Israel Center for Disease Control and the Ministry of Health. 2016).

2. **Fecal sample collection and processing** - Parents were given sterile test tubes (marked for the study participant and the sibling) and written instructions on how to collect and store fecal samples. The parents used sterile swabs to collect approximately 2 grams of fresh stools. The swabs were immediately stored in a home freezer at -20°C. The following day, the test tubes were transported on ice to the hospital and stored at -80°C until they were shipped on dry ice to the Azrieli Faculty of Medicine (Bar-Ilan University, Safed, Israel).

3. **Fecal microbiota transplant to germ-free mice** - GF Swiss Webster male mice were obtained from Taconic Farms Inc. (Germantown, NY, USA) and bred in sterile isolators at the animal facility at the Azrieli Faculty of Medicine. At weaning (4 weeks), the mice were removed from the isolators and transferred to the conventional animal house under a 12 h light–12 h dark regimen with free access to food (1324 TPF, Altromin) and water. Fecal samples from 12 ISS subgroup 2 children and their matched siblings were then transplanted to the GF mice by oral gavage. A second gavage was administered one week later. Each sample was suspended in 800 μl of sterile phosphate-buffered saline and dissolved by vortex for 1 min. A total of 150 μl of the fecal suspension was administered by oral gavage to each pair of mice (150 μl/mouse) who were co-housed in the same cage after the first transplant. The mice were followed for 28 days post-transplant, with day 0 referring to the day of the first fecal transplantation. Weight monitoring and fecal sample collection were performed weekly, starting from day 0 until the end of the experiment at 8 weeks of age. The experiment protocol was approved by the Bar Ilan University Animal Studies Committee (approval number 52-07-2021).

4. **Histological staining and measurement of growth plate height** - After sacrifice, the tibiae and humeri of each animal were carefully removed, cleaned, and measured (length) with a digital caliper. The tibiae and humeri of each mouse were fixed in 10% neutral buffered formalin for 48h at room temperature, decalcified with Surgipath Decalcifier II (Leica Biosystems Richmond, Inc. USA) for several hours, dehydrated through graded ethanol series (70%, 95%, and 100%), and stabilized by 2 sequential changes of chloroform for paraffin embedding. Histological studies and EGP height measurements were performed on paraffin sections of 6μm, and the results were photographed and analyzed by Image-Pro software (version 4.5.1.22, Media Cybernetics, Rockville, MD, USA) as described elsewhere 19. Both bone length and EGP heights were measured by 2 histologists blinded to their origin.
